# Supplementary material for: Patterns of Intron Gain and Loss in Fungi
Source: PLoS Biol. 2004 Nov 30;2(12):e422. doi: 10.1371/journal.pbio.0020422 (PMC532390; doi:10.1371/journal.pbio.0020422)
Supplement: Table S1 — Also available at http://genes.mit.edu/NielsenEtAl/. (4.3 MB ZIP). [file pbio.0020422.st001.zip › NielsenEtAl/html/232.html]

AN0640.1.NCU06465.1.MG06250.1.FG02522.1


```
 CLUSTAL W (1.82) Multiple Sequence Alignments - Introns Inserted


Sequence 1: NCU06465.1	496 aa
Sequence 2: MG06250.1	367 aa
Sequence 3: FG02522.1	373 aa
Sequence 4: AN0640.1	430 aa
Alignment Length: 557 aa
Number Identitical Residues: 159 aa
Alignment Score (without introns) 8939


MG06250.1 	------------------------------------------------------------
NCU06465.1	MAECRWWHDKTADAEERTKAKLMGKNIAALSTWSMMGCPASGILHGFLIFCTKIRQGRMA
FG02522.1 	----------------------MGNDSSILADGSAMVAPG--------------------
AN0640.1  	------------------------------------------------------------
          	                                                            

MG06250.1 	----------------------------------------------------~-------
NCU06465.1	GSRGNSLFSSGSGAANRAKRRTPLDRRTGHGTGDCTRTTKTRQFSTELNSYY2PSMVFTD
FG02522.1 	----------------------------------------------------~-------
AN0640.1  	----------------------------------------------------~-------
          	                                                            

MG06250.1 	---------MANSTVMSDLPPLPSYSLSPLPDLLPFISDFWLPIILPHVAYWMLSLFFHI
NCU06465.1	TATGPSMAMMANTTIAAALPPLPAYETRPLPDLLPWISDFWLSLILPHIAYWVVSMFFHI
FG02522.1 	-------TSFNHTLFETALPPLPIFSLEVQPDLLSWVSDFWLNLLLPVVVYWVLSLTFHA
AN0640.1  	--------MATNTTLLYDLPPLPAYQLTPRPSLVDGIPDNILALILPVVAYWAVSMFFHV
          	           :: .   ***** :.    *.*:  :.*  * ::** :.** :*: ** 

MG06250.1 	IDVYDLFPQYRLHTPDEITSRNHATRTEVLRDVLIQQVIQTITAAVLSATDEPQMVGGED
NCU06465.1	IDVYDLFPQYRLHTPEEISQRNLASRYEVARDVLLEQAIQIGTSAVLNLLDDQQLTGHEN
FG02522.1 	IDVLDLFPQHRLHTPEEITKRNHVSRYEVARDVIVQQVIQVVTGALLALSEPPEMVGKGE
AN0640.1  	IDVYDLFPQYRLHTPAEVLTRNRASRWDVVRDVVLQQVVQTLAGLILAYFDEQEYIGREE
          	*** *****:***** *:  ** .:* :* ***:::*.:*  :. :*   :  :  *  :

MG06250.1 	YAIAVWATRIRLAQRALPTILGVLGLNAAALSKNMASTGHTWLAGALAGGHYPAMSNEFV
NCU06465.1	YEVAVWATRIRLAQRGLPTILGFVGLNAGALSKNIAGS-YPLLAGALAGGFYPSLFELDD
FG02522.1 	YDVAVWATRIRIAQRALPTLLSLVGLNATAISKGLLDS-HPLLAGAIAGGYYPSLVN---
AN0640.1  	YDVAVWARRIRFVQRGLPYLLALFGLDASGVAANFSRNGHKMLAGAVAGGYYPGATQSIV
          	* :**** ***:.**.** :*...**:* .:: .:  ..:  ****:***.**.  :   

MG06250.1 	TG---ATTDAAFAAWELGLAKAIYWFMLPALQFWFAVAFLDTWQYFWHRLMHVNKWMYT1
NCU06465.1	MA---GTAVPAFASWEILLAKAIYWIIIPCIKFWFAAFVLDSWQYLWHRAMHMNKWMYT1
FG02522.1 	-----ANSEPAFASWEITLAKIIYNFMIPAIQYFIGAAIIDTWQYFLHRLMHVNKWLYA1
AN0640.1  	SSGGIETVVPAFTNWEMSLAAFIYWYFIPALQFTWGICVVDTWQYFLHRAMHLNRWLYV1
          	 ...  .  .**: **: **  **  ::*.:::  .  .:*:***: ** **:*:*:*. 

MG06250.1 	NWHSRHHRLYVPYAYGALYNHPIEGFLLDTAGAGLAYKLSTMSPRMGMIFFCFSTIKTVD
NCU06465.1	KWHARHHRLYVPYAYGALYNHPVEGFVLDTLGAAIAYKCSFMTSREGMFFFVGSMMKTVD
FG02522.1 	HFHSRHHRLYVPYAYGALYNHPVEGFLLDTLGAAVAFKVTRMTLRQGILFFSMSTIKTVD
AN0640.1  	HFHSRHHRLYVPYAYGALYNHPVEGLLLDTVGAGIGFLTSGMTHRQSMWFFTFSTIKTVD
          	::*:******************:**::*** **.:.:  : *: * .: **  * :****

MG06250.1 	DHCGYKLPWDPLQRITSNNAAYHDIHHQSWGIKTNFSQPFFTIWDRWLNTMYKGDVTDKY
NCU06465.1	DHCGYALPWDPLQNITSNNAAYHDIHHQSWGIKTNFSQPFFTTWDRILGTMWKGDTSIKY
FG02522.1 	DHCGYAFPWDPLQLVTSNNAEYHDIHHQHYGIKTNFAQPFFTFWDTLLDTKYKGSRSNKP
AN0640.1  	DHCGYAFPWDPLQHATTNNAAYHDIHHQSWGIKTNFSQPFFTFWDRLFNTQWKGEVKLRY
          	***** :******  *:*** ******* :******:***** **  :.* :**. . : 

MG06250.1 	ERAKNAAAN---KAAKTASAKAQ-------------------------------------
NCU06465.1	ERTRAAAANKK-KGAKGEETKAE-------------------------------------
FG02522.1 	SEKK----------AK-QEAKAR-------------------------------------
AN0640.1  	ERSREAAEKKLSEGGMQQDSDMENSSSDFAQPNGEAAVVSPEEPSDRDARTRLRKKTASF
          	.. : :: .. :...   .:. ..:::. :.....::  :....:. .: :   ..::: 

MG06250.1 	-------------------
NCU06465.1	-------------------
FG02522.1 	-------------------
AN0640.1  	SPSVDSLKGVNHGVPSSVL
          	:.: .: .. . . .::
```
